# Proteomic analysis of the pyrenoid-traversing membranes of *Chlamydomonas reinhardtii* reveals novel components

**DOI:** 10.1101/2024.10.28.620638

**Published:** 2024-10-31

**Authors:** Eric Franklin, Lianyong Wang, Edward Renne Cruz, Keenan Duggal, Sabrina L. Ergun, Aastha Garde, Martin C. Jonikas

**Affiliations:** 1 Department of Molecular Biology, Princeton University, Princeton, NJ 08544, USA; 2 Howard Hughes Medical Institute, Princeton University, Princeton, NJ 08544, USA

**Keywords:** Affinity purification, *Chlamydomonas reinhardtii*, CO_2_-concentrating mechanism (CCM), mass spectrometry, membranes, membrane proteins, proteomics, pyrenoid

## Abstract

- Pyrenoids are algal CO_2_-fixing organelles that mediate approximately one-third of global carbon fixation and hold the potential to enhance crop growth if engineered into land plants. Most pyrenoids are traversed by membranes that are thought to supply them with concentrated CO_2_. Despite the critical nature of these membranes for pyrenoid function, they are poorly understood, with few protein components known in any species.
• Here we identify protein components of the pyrenoid-traversing membranes from the leading model alga *Chlamydomonas reinhardtii* by affinity purification and mass spectrometry of membrane fragments. Our proteome includes previously-known proteins as well as novel candidates.
• We further characterize two of the novel pyrenoid-traversing membrane-resident proteins, Cre10.g452250, which we name Pyrenoid Membrane Enriched 1 (PME1), and LCI16. We confirm their localization, observe that they physically interact, and find that neither protein is required for normal membrane morphology.
• Taken together, our study identifies the proteome of pyrenoid-traversing membranes and initiates the characterization of a novel pyrenoid-traversing membrane complex, building toward a mechanistic understanding of the pyrenoid.

## Introduction

Globally, photosynthetic organisms fix over 100 billion metric tons of carbon each year via the conserved enzyme Ribulose-1,5-bisphosphate carboxylase/oxygenase (Rubisco), thought to be the most abundant protein in the world (Ellis, 1979; Field et al., 1998; Beardall & Raven, 2020). Despite its importance to the biosphere, Rubisco runs relatively slowly for an enzyme in central carbon metabolism and is prone to catalyzing oxygenation, a wasteful reaction with abundant oxygen (Flamholz et al., 2019). Some organisms overcome these limitations through the use of CO_2_-concentrating mechanisms (CCMs). These mechanisms improve the efficiency of carbon fixation by concentrating CO_2_ locally around Rubisco to increase its rate of carboxylation and decrease unwanted oxygenation (Hatch, 1987; Lüttge, 2004; Sage et al., 2012; Raven et al., 2017).

Eukaryotic algae, which mediate most of the CO_2_ assimilation in the oceans (Mackinder, 2017), nearly all use a CCM based on a chloroplast-localized organelle called the pyrenoid (Wang et al., 2015; Beardall & Raven, 2020; Meyer et al., 2020a; Barrett et al., 2021; He et al., 2023). A pyrenoid is formed by tightly clustering the cell’s Rubisco into a biomolecular condensate (Freeman Rosenzweig et al., 2017). Concentrated CO_2_ is released inside this condensate, increasing the carboxylation rate of Rubisco and decreasing its oxygenation. Despite their importance to the global carbon cycle, pyrenoids remain understudied and poorly understood at a molecular level.

Because pyrenoids evolved many times across independent algal lineages (Meyer et al., 2020a; Barrett et al., 2021; He et al., 2023), many of the specific genes involved in CCM function differ between phylogenetic groups. However, we expect that common principles of biogenesis and function underlie these convergently-evolved structures. Our approach to uncovering these principles is to study the leading model alga, *Chlamydomonas reinhardtii* (*C. reinhardtii* hereafter), whose pyrenoid is currently best understood. The existence of an extensive experimental toolbox in this organism—including well-defined genetic and proteomic protocols and a library of knockout mutants—makes it a powerful platform for the discovery and characterization of novel pyrenoid components.

The *C. reinhardtii* pyrenoid consists of a phase-separated matrix containing tightly-packed Rubisco (Freeman Rosenzweig et al., 2017), surrounded by a starch sheath that appears to limit CO_2_ leakage (Toyokawa et al., 2020; Fei et al., 2022). Traversing the matrix are specialized membranes that are thought to mediate the delivery of concentrated CO_2_ to the matrix (Raven, 1997; Karlsson et al., 1998; Sinetova et al., 2012; Fei et al., 2022).

In *C. reinhardtii* the pyrenoid-traversing membranes form a network of tubules that is contiguous with but ultrastructurally distinct from the photosynthetic thylakoid membranes (Engel et al., 2015). Tubules are constitutively present including during cell division (Goodenough, 1970), in non-CCM-requiring growth conditions (Ma et al., 2011; Caspari et al., 2017), and in mutants deficient in pyrenoid matrix formation (Goodenough & Levine, 1970; Ma et al., 2011; Mackinder et al., 2016; Caspari et al., 2017; He et al., 2020). The tubules are structurally complex, being composed of a central reticulated region with three-way junctions out of which radiate discrete cylindrical tubules, which themselves contain smaller minitubules that are apparently also made of membranes (Engel et al., 2015). The tubules are organizationally dynamic, with tubule quantity and diameter varying under different growth conditions (Rawat et al., 1996). Given the presence of the tubules in the absence of a pyrenoid matrix, it has been proposed that they play an organizational role in pyrenoid nucleation or localization (Meyer et al., 2020b), while their presence in non-CCM-requiring growth conditions suggests possible additional functions beyond CO_2_ delivery.

To date, several tubule-localized proteins have been identified with varying degrees of confidence. The best-established proteins that localize specifically to the tubules are Carbonic AnHydrase 3 (CAH3; Cre09.g415700), which is thought to deliver CO2 to the pyrenoid; MIssing THylakoids 1 (MITH1; Cre06.g259100), which is necessary for producing pyrenoid-traversing membranes (Hennacy et al., 2024); and Rubisco-Binding Membrane Protein 1/Bestrophin 4 (RBMP1/BST4, Cre06.g261750) and RBMP2 (Cre09.g416850), whose functions remain poorly characterized (Meyer et al., 2020b).

Multiple strategies have been employed to isolate and identify *C. reinhardtii* pyrenoid proteins, including fractionation of whole pyrenoids (Mackinder et al., 2016; Zhan et al., 2018), systematic protein localization and affinity purification of protein complexes using known pyrenoid components as baits (Mackinder, 2017; Wang et al., 2023), and proximity labeling of pyrenoid proteins using TurboID (Lau et al., 2023).

Here, we identify tubule-localized proteins by developing a complementary approach that relies on isolating fragments of pyrenoid-traversing membranes. We show that the approach successfully recovers known tubule proteins and identifies novel candidates. We further validate and characterize two of the top candidates. By expanding the known pyrenoid tubule proteome, this work contributes to paving the way toward a mechanistic understanding of pyrenoid function.

## Materials and Methods

### Strains and Culture Conditions

All insertional mutants were obtained from the CLiP collection at the Chlamydomonas Resource Center (Li et al., 2019). Mutant IDs for these strains can be found in Table 1 below. The parent strain for these mutants is cMJ030 (CC-4533) and is also available from the Chlamydomonas Resource Center.

Unless otherwise noted, cultures were grown in TP media to a concentration of ~2 × 10^6^ cells/mL prior to experiments. Unless otherwise noted, cultures were grown in low CO_2_ (air, ~0.04% v/v CO_2_) and 150 μmol · m^−2^ · s^−1^ photons.

All strains generated in this work were deposited to the Chlamydomonas Resource Center (https://chlamycollection.org).

### Membrane Affinity Purification and Mass Spectrometry

Detergent-based affinity purification of and mass spectrometry of RBMP1-Venus-3×FLAG, RBMP2-Venus-3×FLAG, and LCI16-Venus-3×FLAG were performed as described previously (Wang et al., 2023).

For the membrane affinity purification, our objective was to isolate fragments of membranes of interest. We sought to achieve this by choosing baits localized to our membranes of interest and performing a standard affinity purification, as described in (Wang et al., 2023), but with the membrane disruption step changed from dissolution with detergent to shearing with sonication. We used two bait proteins which localize to the tubules, RBMP1 (Cre06.g261750) and RBMP2 (Cre09.g416850) (Meyer et al., 2020b), and two bait proteins for the thylakoids, CPLD9 (Cre01.g005001) and CGL129 (Cre05.g233950) (Wang et al., 2023). We performed three iterations of this membrane affinity purification experiment, each with slight differences meant to better enrich membranes or detect the proteins enriched in the purification.

For the first membrane affinity purification, all steps—including cell growth, affinity purification, and mass spectrometry—were performed as in (Wang et al., 2023), with the following changes: 1) any buffer with more than 0.1% digitonin was prepared without digitonin; 2) In place of membrane solubilization using IP buffer + digitonin followed by centrifugation at 12,700 × g, samples were instead sonicated with a probe sonicator (QSonica) in 20 cycles (5 × 1 sec. pulse, 30 sec. rest) in a microcentrifuge tube on ice followed by centrifugation at 1000 × g before progressing to incubation with anti-FLAG beads; 3) After elution and denaturation, samples were loaded into a 4-20% Criterion TGX Precast Mini Protean Gel (BioRad) for electrophoresis at 50 V for 40 min until the protein front moved ~2.0 cm to separate the FLAG peptides from the eluted proteins.

The second affinity purification replicated the first with the following additional changes: 1) Sonicated lysate was centrifuged at 5,000 × g; 2) In place of elution with FLAG peptide and in-gel digestion of proteins, proteins were digested on-bead using S-Trap^™^ Micro Spin Column Digestion (Protifi); 3) Samples were run on the mass spectrometer for 2 hours instead of 1 hour.

The third affinity purification was performed like the first with the following changes: 1) sonicated lysate was centrifuged at 5,000 × g; 2) after elution, samples were frozen and submitted to the Thermo Fisher Scientific Center for Multiplexed Proteomics at Harvard Medical School for digestion, tandem mass tag (TMT) labeling (Thermo Fisher), and quantitative mass spectrometry (Ting et al., 2011; McAlister et al., 2012; Li et al., 2020).

The full results for all three affinity purification experiments can be found in Supporting Information Tables S1-3.

### Live-Cell Imaging

For live-cell imaging, strains expressing Venus-tagged proteins were grown at 3% CO_2_ in TP media to a concentration of ~ 5 × 10^5^ cells mL^−1^, then transferred to ambient CO_2_ 16 hours before collecting for imaging at a concentration of ~1-2 × 10^6^ cells mL^−1^. 200 μL of each cell culture were placed into the wells of an 8-well μ-slide (Ibidi) and allowed to settle for 5 minutes. The culture media was aspirated to leave a thin layer of cells, and the cells were immobilized for imaging by flowing over 1 mL of 2% low-melting-point TP agarose in each well at ~40°C, which was allowed to set for 5 minutes at room temperature prior to imaging.

Cells were imaged using a VT-iSIM super-resolution spinning-disk confocal microscope on an Olympus iX83 body equipped with VisiView software, a 60X 1.43 NA objective and a Hamamatsu Orca Quest sCMOS camera in slow mode. Venus fluorescence was excited using a 514 nm laser and collected at 545/50 nm. Chlorophyll autofluorescence was excited using a 642 nm laser and collected at 700/75 nm. Confocal z-series were acquired with a 300 nm Z-step. The same acquisition settings were used for all strains imaged in this study. Images were imported into Fiji (Schindelin et al., 2012) for deconvolution using the Microvolution plugin and final image preparation.

### Transmission Electron Microscopy (TEM)

TEM was performed as described in (He et al., 2020) with some modifications. Strains for TEM imaging were grown at 3% CO_2_ in TP media to a concentration of ~5 × 10^5^ cells mL^−1^, then transferred to ambient CO_2_ 16 hours before harvesting at a concentration of ~1-2 × 10^6^ cells mL^−1^. At least 50 × 10^6^ cells were harvested by centrifugation at 1,000 × g for 5 minutes in 50 mL tubes, resuspended in 1 mL TP, transferred to 1.5 mL screw-top tubes, and centrifuged again for 5 minutes at 1,000 x g. Cells were then resuspended in 2.5% gluteraldehyde in TP media (10 mL 10% gluteraldehyde, 30 mL TP) and nutated at room temperature for 1 hour. After nutation, cells were pelleted at 3,000 × g for 1 minute, then washed 3 times by resuspending and nutating in 1 mL ddH_2_O for 5 minutes each, pelleting at 3,000 × g after each wash. Samples were then post-fixed and stained with 1 mL freshly-prepared osmium tetroxide solution (1% OsO_4_, 1.5% w/v K_3_[Fe(CN)_6_], 2 mM CaCl_2_) and nutated for 1-2 hours. Tubes were wrapped in aluminum foil during nutation to protect samples from light. Counterstaining with uranyl acetate was found to increase background staining and reduce contrast when looking at membranes, so for all images shown and analyzed here were stained using only OsO_4_. Samples were then serially dehydrated by resuspension and nutation in increasing concentrations of ethanol: 5 minutes in 1 mL each of 30%, 50%, 70%, and 95% ethanol, then 10 minutes in 100% ethanol and 2 × 10 minutes in 100% acetonitrile. Samples were pelleted for 1 minute at 3,000 × g between each resuspension.

After dehydration, samples were embedded in epoxy resin containing 34% Quetol 651, 44% nonenyl succinic anhydride, 20% methyl-5-norbornene-2,3-dicarboxylic anhydride and 2% catalyst dimethylbenzylamine (Electron Microscopy Sciences) over four days, first by embedding overnight in 1:1 acetonitrile:resin (no catalyst) with the screw-top tube open in a fume hood, then by nutating in 1 mL Quetol resin (including catalyst) for four days, spinning down the samples and resuspending in fresh resin once per day. On day 4, the samples were resuspended in 300-500 μL resin, centrifuged at max speed (18,213 x g) for 20 minutes at 30°C in a tabletop centrifuge with a swinging bucket rotor for microfuge tubes, then cured at 60–65°C for 48 hours.

Thin (~70 nm) sections were prepared from cured resin blocks on a Leica Microtome Ultracut UCT and mounted on carbon film–coated 200 mesh copper TEM grids (Electron Microscopy Sciences) and imaged at the Imaging and Analysis Center, Princeton University, using a CM200 TEM (Philips) or a Talos F200X STEM (ThermoFisher Scientific).

## Results

### We used affinity purification to isolate membrane fragments

The use of affinity purification to identify protein complexes is well-documented (Morris et al., 2014; Meyer & Selbach, 2015). Such methods usually involve the use of detergents during cell lysis to dissolve membranes (Behnke & Urner, 2023), such that proteins normally only co-precipitate with the bait protein if they are part of the same protein complex. This strategy has been used to identify novel protein complexes and elucidate functions for previously uncharacterized genes in Chlamydomonas (Mackinder, 2017; Wang et al., 2023). However, here we wanted to isolate fragments of tubule membrane, with the hopes that we could then comprehensively identify pyrenoid tubule-localized proteins.

**Figure 1:**
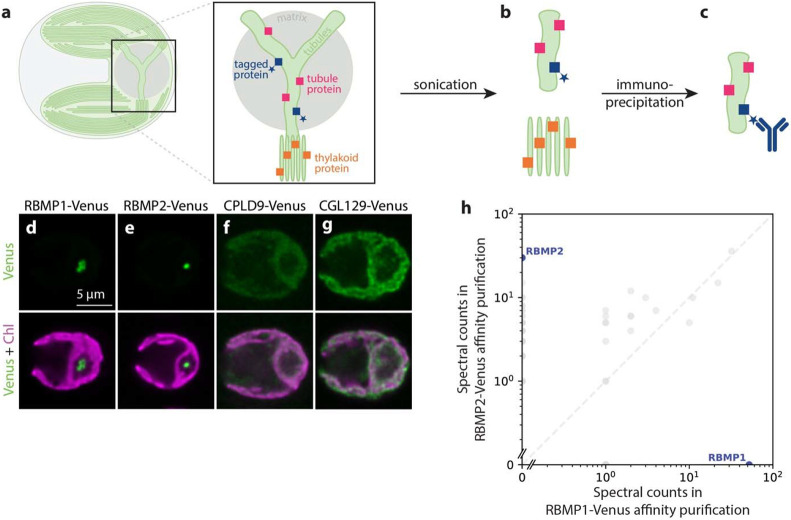
We adapted an affinity precipitation protocol to purify intact membranes. **a–c.** Schematic of the membrane affinity purification. **a.** Cartoon representation of a *C. reinhardtii* cell, with the pyrenoid in the base of the cup-shaped chloroplast. Inset: The pyrenoid is traversed by tubule membranes which are contiguous with but structurally distinct from the photosynthetic thylakoids. Colored squares represent the proteins localized to the membranes: blue squares with a star represent known tubule proteins tagged with a FLAG tag, pink squares represent all other proteins localized to the tubules and potentially pulled down by our experiment, and orange squares represent proteins mainly localized in the thylakoid membranes. **b.** By disrupting cellular membranes using sonication, we sought to create small membrane fragments whose protein composition was maintained. **c.** Membrane fragments containing tagged proteins were immunoprecipitated using an anti-FLAG antibody and their protein composition was determined by mass spectrometry. **d–g.** Confocal fluorescence images showing the localization of the FLAG-tagged proteins used as baits for the membrane affinity purification. Chlorophyll, a proxy for the presence of thylakoid membranes, is shown in magenta. The pyrenoid is in the low-chlorophyll region in the base of each cell. Venus signal is shown in green, representing the localization of the tubule **(d–e)** or thylakoid **(f–g)** bait proteins. **h.** Results of a detergent-based affinity purification show that the tubule bait proteins RBMP1-Venus and RBMP2-Venus do not co-precipitate using traditional affinity purification methods.

We therefore adapted an existing affinity purification protocol used in *C. reinhardtii* (Mackinder, 2017; Wang et al., 2023) to affinity purify intact membrane fragments. Using tagged membrane proteins of known localization (Fig. 1a) as bait, we performed the affinity purification protocol on cell lysate in which membranes were disrupted by sonication rather than solubilized by detergent (Fig. 1b). We reasoned that this approach would produce membrane fragments that would allow the identification of proteins that co-localized to the same regions of membranes even if they did not directly interact physically (Fig. 1c).

We selected four bait proteins on which to perform this protocol: RBMP1-Venus-3×FLAG and RBMP2-Venus-3×FLAG (Fig. 1d,e), which localize to the pyrenoid tubules (Meyer et al., 2020b); and CPLD9-Venus-3×FLAG and CGL129-Venus-3×FLAG (Fig. 1f,g), which localize to the thylakoid membranes and are excluded from the tubules (Wang et al., 2023). RBMP1 and RBMP2 localize to distinct regions of the tubules—RBMP1 to the tubule periphery and RBMP2 to the central reticulated region (Meyer et al., 2020b)—thus, we reasoned that together they should provide coverage for the whole tubule network. As expected from their distinct localizations, RBMP1 and RBMP2 did not co-precipitate via traditional detergent-based affinity purification (Fig. 1h, Supporting Information Table S4).

### Identification of a pyrenoid-traversing membrane proteome

**Figure 2:**
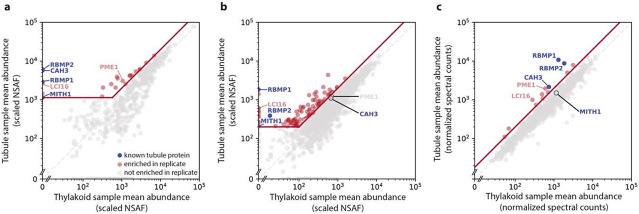
The membrane affinity purification experiment identified both known and novel tubule proteins. Protein abundances in tubule samples vs. thylakoid samples for the three membrane affinity purification experiments. Blue points represent the known tubule proteins RBMP1, RBMP2, CAH3, and MITH1. The grey dashed lines represent y=x, at which proteins are equally abundant in both the tubule and thylakoid samples. The diagonal red line represents 2-fold enrichment in the tubule samples. The first two experiments **(a–b)** were quantified using label-free quantification, leading to significant noise among lower-abundance proteins. For these experiments, hits (red points) were defined as exceeding 2-fold enrichment in the tubule sample and exceeding a lower abundance threshold (horizontal red line). Proteins in the third membrane affinity purification **(c)** were quantified using tandem mass tagging, leading to better quantification of low abundance proteins. Thus, only 2-fold enrichment was required to be considered a hit. Proteins that are enriched in at least 2 experiments can be found in Table 3.

We performed three iterations of the membrane affinity purification experiment, each with slight changes to the protocol to try and improve the enrichment or identification of membrane proteins. Samples from the first two experiments were quantified using label-free quantification (LFQ), identifying 58 and 135 proteins that were at least 2-fold enriched in the tubule samples relative to the control samples (Fig. 2a,b, Supporting Information Tables S1-S2). Quantification using LFQ, however, can suffer from Poisson noise for lower-abundance proteins, leading some low-abundance proteins to appear enriched or depleted in a given sample due to differences in random run-to-run peptide sampling rather than differences in actual abundance in each sample (O’Connell et al., 2018). We therefore set a minimum abundance threshold for proteins to be considered hits in those experiments, which reduced the number of 2-fold-enriched hits in the first two experiments to 22 and 92, respectively. We performed a third iteration of the membrane purification experiment using tandem mass tag (TMT) labeling to better quantify lower-abundance proteins in our samples. This TMT experiment yielded 14 proteins that were at least 2-fold enriched in the tubule samples (Fig. 2c, Supporting Information Table S3).

The highest-confidence hits from the membrane affinity purification experiments are listed in Table 1. These highest-confidence hits were selected on the basis of being 2-fold enriched in the tubule samples in at least two of our three experiments. Four previously-known tubule proteins were among these proteins: the two bait proteins, RBMP1 and RBMP2, the carbonic anhydrase CAH3 (Sinetova et al., 2012), and MITH1 (Hennacy et al., 2024).

Less-highly-enriched were proteins such as HCF136 (Cre06.g273700) and TEF14 (Cre06.g256250), which are photosystem or photosystem-associated components that have been shown to be enriched in, but not exclusively localized to, the pyrenoid tubules (Mackinder, 2017; He et al., 2023; Wang et al., 2023), or CAS1 (Cre12.g497300 ), a calcium-sensing protein which is found throughout the chloroplast under high CO_2_ but relocalizes to the tubules under the growth conditions used in our study (Wang et al., 2016) (Supporting Information Tables S1-S3).

Importantly, our results are consistent with the enrichment of proteins based on membrane proximity rather than direct protein-protein interaction, as we were able to identify proteins not seen in traditional detergent-based affinity purification. Neither MITH1 nor CAH3 co-immunoprecipitated with RBMP1 or RBMP2 in a detergent-based affinity purification (Supporting Information Table S4), yet both are enriched in our tubule samples (Fig. 2). The presence of CAH3, a pyrenoid tubule-enriched luminal protein with no predicted transmembrane domains (Sinetova et al., 2012), also suggests that we are able to capture proteins within the membrane lumen. Furthermore, RBMP1 and RBMP2 did not co-precipitate in detergent-based affinity purification (Fig. 1h, Supporting Information Table S4), whereas they did co-precipitate in our first and third membrane purifications (Table 3). Taken together, these results indicate that our protocol is able to isolate and identify tubule membrane proteins that are not captured by traditional affinity purification approaches.

### Identification of novel candidate tubule proteins

In addition to the previously-known tubule proteins, the top hits included two novel candidate tubule proteins: Low-CO_2_-induced 16, also known as Early Light-Induced 4 (LCI16/ELI4; Cre02.g143550) and Cre10.g452250. LCI16 has a chlorophyll-binding domain which is homologous to light-harvesting complex–like proteins present across plant and algal species (Rochaix & Bassi, 2019). These proteins are often induced by various abiotic stresses such as high light, high salinity, and high temperature; and function in photoprotection and assembly/repair of photosystems (Montané & Kloppstech, 2000; Rochaix & Bassi, 2019). Consistent with a potential function in tubules, LCI16 showed a similar pattern of diurnal mRNA expression to those of other tubule proteins, including RBMP1, RBMP2, and SAGA1, with a peak right after dawn (Supporting Information Fig. S1) (Strenkert et al., 2019).

Cre10.g452250, which we named Pyrenoid Membrane-Enriched 1 (PME1), does not have any homologs we could identify. However, it does have a putative PRICHEXTENSN-like domain (Showalter et al., 2010; Guo et al., 2014) (Goodstein et al., 2012; Craig et al., 2023), a poorly-characterized proline-rich domain also found in RBMP1 and RBMP2. Additionally, although its expression levels were lower, PME1 also showed a peak in mRNA levels at or just before dawn, similar to LCI16, RBMP1, RBMP2, and SAGA1 (Supporting Information Fig. S1) (Strenkert et al., 2019), further supporting a potential function in pyrenoid tubules.

Given the presence of LCI16 and PME1 in our tubule proteome and the similarities they share with other known tubule proteins, we decided to further characterize these novel tubule candidates to validate their tubule localization and determine if they impact pyrenoid tubule biogenesis.

### Fluorescently-tagged LCI16 localizes to the pyrenoid-traversing membranes

To confirm that our novel tubule candidates are localized to the pyrenoid tubules, we created constructs to express each gene tagged with a Venus fluorophore and transformed each construct into wild-type cells (CC-4533). Cells transformed with PME1-Venus-3×FLAG constructs consistently showed antibiotic resistance but failed to express detectable levels of PME1-Venus-3×FLAG, possibly due to the lower expression levels of PME1 (Strenkert et al., 2019). Thus, we focused our efforts on characterizing LCI16-Venus-3×FLAG transformants, which yielded detectable fluorescence.

**Figure 3:**
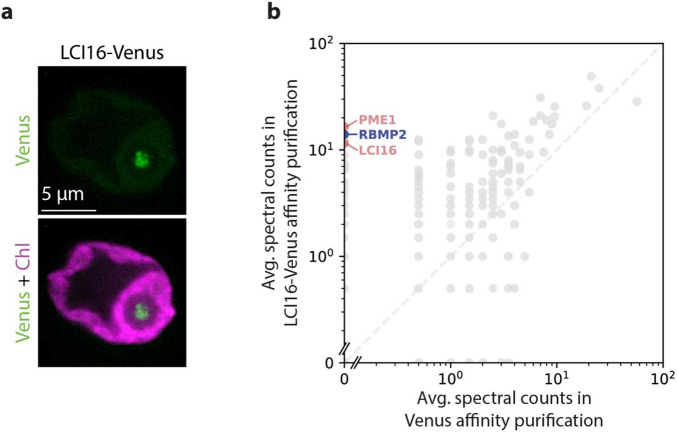
LCI16-Venus localizes to the pyrenoid tubules and co-precipitates RBMP2 and PME1. **a**. Confocal images showing the localization of LCI16-Venus in the pyrenoid tubules. Green represents LCI16-Venus signal and magenta represents chlorophyll. **b**. Average spectral counts for a detergent-based affinity purification of LCI16-Venus compared to a control affinity purification of Venus alone. The blue point represents the known tubule protein RBMP2, while the red points represent the novel tubule components LCI16 and PME1.

We observed via fluorescence microscopy that LCI16-Venus-3×FLAG localizes to the center of the pyrenoid (Fig. 3a). Its localization pattern was similar to those of RBMP1-Venus-3×FLAG and RBMP2-Venus-3×FLAG (Fig. 1d,e) in being more compact and more heterogeneous than matrix-localized proteins such as Rubisco (Hennacy et al., 2024). Based on this localization pattern and LCI16’s enrichment in our pyrenoid-traversing membrane proteome (Fig. 2 and Table 3), we conclude that LCI16 localizes to pyrenoid-traversing membranes.

### LCI16 physically interacts with RBMP2 and PME1

To further investigate the role of LCI16 in the tubule membranes, we performed a traditional detergent-based affinity purification using LCI16-Venus-3×FLAG as bait to determine what proteins it directly interacts with (Fig. 3b, Supporting Information Table S5). In this experiment, the top two interactors of LCI16 were PME1 and RBMP2, indicating that these three proteins directly or indirectly physically interact in one or more protein complexes and providing additional support for PME1’s tubule localization.

Our previous detergent-based RBMP1-Venus-3×FLAG and RBMP2-Venus-3×FLAG affinity purifications did not identify LCI16 or PME1 as highly enriched (Supporting Information Table S4). However, previously-published proteomic data from diurnally-grown cells suggest that RBMP2 may be more abundant than LCI16 or PME1 (Supporting Information Table S1) (Strenkert et al., 2019). This raises the possibility that a large proportion of LCI16 and PME1 may be bound to RBMP2 even while the majority of RBMP2 is not bound to LCI16 or PME1. Unfortunately, due to our inability to produce a PME1-Venus-3×FLAG strain, we were unable to affinity purify PME1 to determine if all three proteins form a single complex or if LCI16 interacts with RBMP2 and PME1 separately.

**Figure 4:**
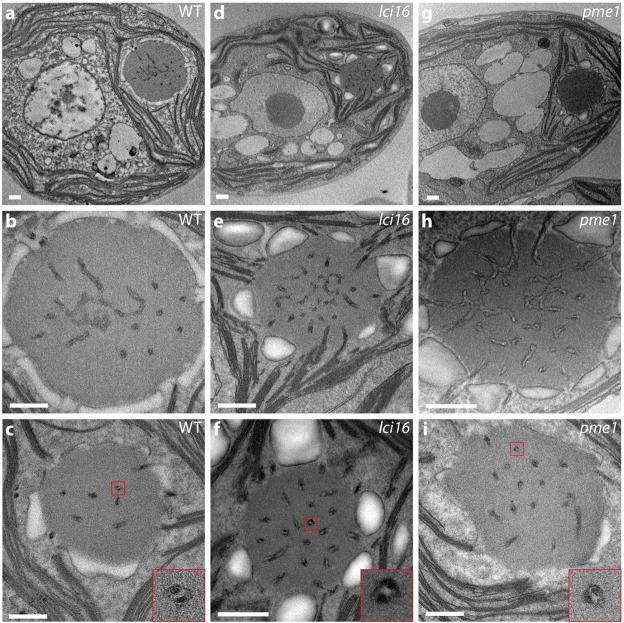
Transmission electron microscopy reveals normal pyrenoid morphology in insertional mutants of *lci16* and *pme1*. Electron micrographs of wild type **(a–c)**, *lci16* **(d–f)**, and *pme1* **(g–i)** cells. Images of central slices of whole cells **(a,d,g)** show the single pyrenoid in its stereotyped location at the base of the cup-shaped chloroplast. Higher-magnification images of the pyrenoids **(b–c,e–f, h–i)** show tubules in all of the pyrenoids, including both the central reticulated region, characterized by three-way membrane junctions **(b,e,h)** and the peripheral minitubule-containing tubules **(c,f,i)**. 4x insets in **(c,f,i)** show individual tubules with visible minitubules. Pyrenoids in **(b,e,h)** represent the same cells as shown in **(a,d,g)**. Scale bars 500 nm.

**Transmission electron microscopy (TEM) of insertional mutants in LCI16 and PME1** To assess the potential role(s) of LCI16 and PME1, we examined pyrenoid morphology in mutant strains lacking expression of each protein using transmission electron microscopy (TEM). We confirmed the insertion sites in one *lci16* and three *pme1* mutants by PCR (Supporting Information Fig. S2) and examined all mutants by electron microscopy. Unlike *mith1* and *saga1* mutants, which have the most-severe tubule-related defects observed in *C. reinhardtii* to date—with most Rubisco condensates entirely lacking tubules (Hennacy et al., 2024)—the *lci16* and *pme1* mutants examined by TEM showed wild-type pyrenoid morphology. We regularly observed only one pyrenoid per cell, and we could observe central reticulated regions (Fig. 4b,e,h) and peripheral cylindrical tubules with minitubules (Fig. 4c,f,i, Supporting Information Figure S3) that were indistinguishable from those in wild-type cells. These results indicate that LCI16 and PME1 are not required for normal tubule morphology under our culture conditions, and suggest that they function in processes unrelated to tubule biogenesis.

## Discussion

In this study, we enriched pyrenoid-traversing membrane fragments from *C. reinhardtii* and identified their associated proteins. From this dataset, we further characterized two novel tubule protein candidates, LCI16 and PME1.

Our membrane affinity purification approach overcame the challenge that pyrenoid tubules are contiguous with—and vastly less abundant than—the thylakoid membranes, impeding their isolation by traditional membrane fractionation methods (Bailyes et al., 1997; Avila et al., 2015). Our protocol overcame this challenge by enriching specific membrane subdomains based on the presence of a known resident protein, allowing us to identify novel protein components of the chosen membrane starting with only the identity of two of its protein components. The limitations of our implementation included low-number sampling noise due to analysis of the first two experiments by label-free mass spectrometry, which limited our ability to detect lower-abundance proteins; and changes to the protocol over the three iterations, which we think contributed to variability in hits between the three iterations. With further improvements, we believe our approach could be applied to determine the protein composition of other membrane domains on a sub-organellar level.

One of the proteins identified by our protocol is Low-CO_2_-Induced 16 (LCI16), also known as Early Light-Induced 4 (ELI4). LCI16 has a LHC-like domain homologous to those found in the stress-related LHCs (LHCSR) and Early Light-Induced Protein (ELIP) families, which are characterized by a 3-helix chlorophyll-binding domain (Rochaix & Bassi, 2019; Levin & Schuster, 2023). These proteins are often induced by dark-to-light transitions, high light, and/or other abiotic stresses, and are typically involved in photoprotection via non-photochemical quenching (NPQ) as well as assembly of new photosystems or repair of damaged photosystems (Montané & Kloppstech, 2000; Rochaix & Bassi, 2019; Levin & Schuster, 2023). Given the localization of photosystem assembly factors to the periphery of the pyrenoid tubules (Mackinder, 2017) and its expression pattern (Strenkert et al., 2019), LCI16 may assist in the synthesis of new photosystems in or near the pyrenoid. Future work will be needed to elucidate its precise role.

The other novel tubule protein we identified and characterized, PME1, interacts with LCI16 and possibly RBMP2. PME1 shares a predicted poorly-characterized PRICHEXTENSN domain with RBMP1 and RBMP2. PRICHEXTENSN domains are found in plant extensins, a class of proteins associated with cell wall stability in plants. Plant extensins are typically characterized by PRICHEXTENSN domains made up of SP_3-4_ repeats (Guo et al., 2014), whereas in PME1 these repeats are even more highly-enriched in proline residues, containing SP_5-7_ repeats interspersed with (A/R/F)P_2_ repeats. Given the presence of this domain in multiple tubule proteins, it may play a role in tubule structure, function, or protein targeting to tubules. If the PRICHEXTENSN-like domains of PME1, RBMP1, and RBMP2 are partially redundant, generation of double or triple mutants among PME1, RBMP1, and RBMP2 may reveal phenotypes that would not be seen in the single mutants.

Interestingly, Alphafold3 (Abramson et al., 2024) predicts that PME1’s C-terminus contains a long amphipathic helix (Supporting Information Fig. S4). The structure prediction indicates this amphipathic helix may be involved in forming a homo-trimer, though this may be an artefact of Alphafold3’s assumption that folding is taking place in an aqueous environment with no membranes. In addition to oligomerization (Truebestein & Leonard, 2016), amphipathic helices are also common among several families of membrane-binding and -shaping proteins, such as BAR domain proteins (Gallop et al., 2006; Jao et al., 2010) and reticulons (Brooks et al., 2021), suggesting a potential mechanism for membrane binding in the C-terminal domain of PME1. Future work is needed to determine if this amphipathic helix is responsible for oligomerization or membrane binding.

Taken together, our results identify LCI16 and PME1 as novel pyrenoid-traversing membrane components, initiate their characterization, and suggest directions for future research into their functions. Our pyrenoid-traversing membrane proteome identifies known components and additional candidates, helping to prioritize proteins for future study. We hope these findings will ultimately contribute toward a mechanistic understanding of the pyrenoid, an organelle with a central role in the global carbon cycle.

## Supplementary Material

Supplement 1

Supplement 2

Supplement 3

Supplement 4

Supplement 5

Supplement 6

## Figures and Tables

**Table 1.** Strains used in this work.

Chlamydomonas Resource Center ID	Strain Description	Mating Type *(mt)*	Source	Antibiotic Resistance
CC-4533	Wild type CMJ030	*mt(−)*	Wild type and parent strain to CLiP library (Zhang et al., 2014)	none
CC-5415	Wild type	*mt(+)*	Wild type and parent strain to *lci16-2, lci16-3*, and *lci16-4*	none
CC-5646	CMJ030;RBMP1-Venus-3×FLAG	*mt(−)*	Previously published (Meyer et al., 2020b)	Paromomycin
CC-5647	CMJ030;RBMP2-	*mt(−)*	Previously published	Paromomycin
	Venus-3×FLAG		(Meyer et al., 2020b)	
CSI_LW07G9	CMJ030;CPLD9-Venus-3×FLAG	*mt(−)*	CSI collection (Wang et al., 2023)	Paromomycin
CSI_LW01C3	CMJ030;CGL129-Venus-3×FLAG	*mt(−)*	CSI collection (Wang et al., 2023)	Paromomycin
	CMJ030;LCI16-Venus-3×FLAG	*mt(−)*	CMJ030 transformed with pLM005-LCI16-Venus-3×FLAG	Paromomycin
LMJ.RY0402.141937	*pme1-1*	*mt(−)*	CLiP library (Li et al., 2019)	Paromomycin
LMJ.RY0402.168140	*pme1-2*	*mt(−)*	CLiP library (Li et al., 2019)	Paromomycin
LMJ.RY0402.236076	*pme1-3*	*mt(−)*	CLiP library (Li et al., 2019)	Paromomycin
LMJ.RY0402.215307	*lci16*	*mt(−)*	CLiP library (Li et al., 2019)	Paromomycin

**Table 2.** Primers used for PCR verification of insertional mutants

Primer name	Sequence
TS.Hb.For	CGGTGATACTTACACGCCC
TS.Hb.Rev	CACAGTTTGTGTGGAATCGG
TIP.Q.For	CAGGGGAGTAGCAAAACAG
TIP.Q.Rev	GCTGCTTCATGTGACCTTG
TIP.Z4.For	CATAGAGCTTGCCATGTTATATCC
TIP.Z4.Rev	AGAAGGAGACGAAAGCACAGG
TIP.Z2/3.For	GGCTTGGATGACTGATGACCG
TIP.Z2/3.Rev	ACCTCGCCAATGCACAGACG

**Table 3.** Proteins enriched in at least two membrane affinity purification experiments Spectral counts in each affinity purification sample for the proteins identified as enriched in at least two out of the three membrane affinity purification experiments. These values are scaled normalized spectral abundance factor values for experiments 1 and 2, which were quantified using label-free quantification; and normalized spectral counts for experiment 3, which was quantified using tandem-mass-tagging. The names of previously-known tubule proteins are bolded.

		Experiment 1					Experiment 2					Experiment 3					
Locus	Gene Name	RBMP1	RBMP2	CPLD9	CGL129	fold enriched	RBMP1	RBMP2	CPLD9	CGL129	fold enriched	RBMP1	RBMP2	CPLD9	CGL129	fold enriched	enriched
Cre06-g261750	RBMP1	5548	281	0	0	INF	3678	0	0	0	INF	19792	1762	1159	1446	8.3	3
Cre09.g416850	RBMP2	3669	8470	0	0	INF	50	727	0	36	21.9	3429	13989	1818	1860	4.7	3
Cre02.g143550	LCI16	3312	2012	0	0	INF	908	312	0	0	INF	2066	710	478	504	2.8	3
Cre09.g415700	CAH3	5935	5454	0	0	INF	1642	565	549	772	1.7	2955	1346	702	777	2.9	2
**Cre06.g259100**	MITH1	704	1569	0	0	INF	0	399	0	0	INF	1640	1370	1173	1118	1.3	2
Cre10.g452250	PME1	1454	4711	1196	906	2.9	798	1646	801	563	1.8	1677	2240	651	555	3.2	2

## Data Availability

The data that supports the findings of this study are available in Supporting Information Tables S1-5 included with this article.
