# Supplementary material for: Proteomic analysis of the pyrenoid-traversing membranes of Chlamydomonas reinhardtii reveals novel components
Source: bioRxiv. 2024 Oct 31:2024.10.28.620638. Preprint. [Version 1] doi: 10.1101/2024.10.28.620638 (PMC11565738; doi:10.1101/2024.10.28.620638)
Supplement: Supplementary file 1 [file NIHPP2024.10.28.620638v1-supplement-6.pdf]

bioRxiv preprint doi: https://doi.org/10.1101/2024.10.28.620638; this version posted October 31, 2024. The copyright holder for this preprint (which was not certified by peer review) is the author/funder, who has granted bioRxiv a license to display the preprint in perpetuity. It is made available under aCC-BY 4.0 International license.

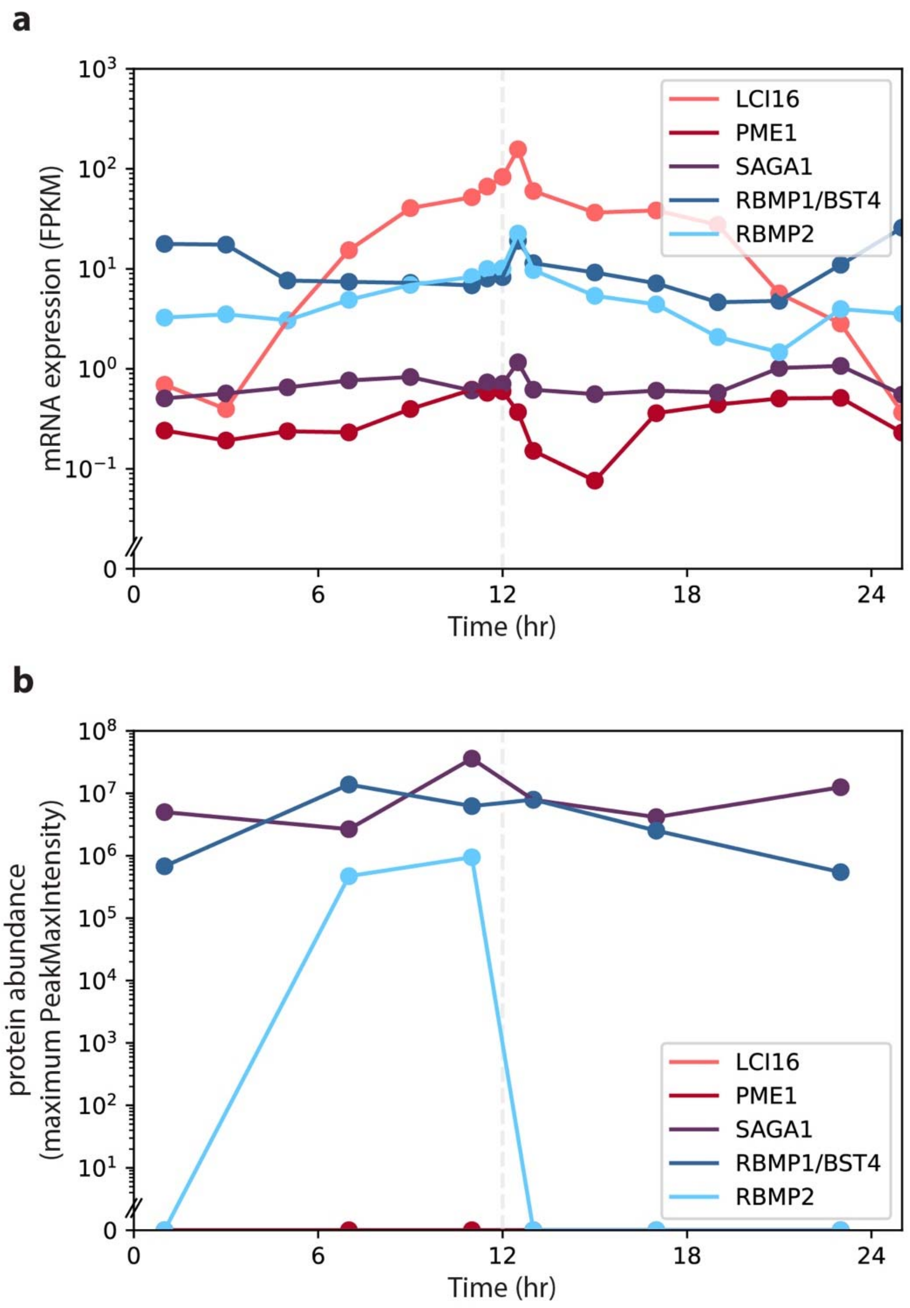

Supporting Information Figure S1: Transcript and protein levels

Transcriptomic and proteomic data from diurnally-grown *C. reinhardtii* cells measuring the abundance of mRNA (**a**) and protein (**b**) levels of the known pyrenoid tubule proteins RBMP1, RBMP2, and SAGA1 (blue/purple lines) compared to the novel tubule components LCI16 and PME1 (red/pink lines). Cells were synchronized and grown in a 12-hour light/12-hour dark cycle, with hour 12 corresponding to the onset of light. Based on data reported in (Strenkert *et al.*, 2019). **a.** mRNA levels of known and novel tubule proteins throughout the diurnal cell cycle.

bioRxiv preprint doi: https://doi.org/10.1101/2024.10.28.620638; this version posted October 31, 2024. The copyright holder for this preprint (which was not certified by peer review) is the author/funder, who has granted bioRxiv a license to display the preprint in perpetuity. It is made available under aCC-BY 4.0 International license.

Baseline mRNA levels differ for each gene, but each expression pattern shows a peak at (PME1) or directly after (RBMP1, RBMP2, SAGA1, LCI16) the onset of light. **b.** Protein abundance of known and novel tubule proteins throughout the diurnal cell cycle. The novel tubule proteins LCI16 and PME1 are not detected at any time point, while the known tubule proteins RBMP1, RBMP2, and SAGA1 are all detected at low levels, either consistently throughout the cell cycle (RBMP1 and SAGA1) or only in the dark (RBMP2)

bioRxiv preprint doi: https://doi.org/10.1101/2024.10.28.620638; this version posted October 31, 2024. The copyright holder for this preprint (which was not certified by peer review) is the author/funder, who has granted bioRxiv a license to display the preprint in perpetuity. It is made available under aCC-BY 4.0 International license.

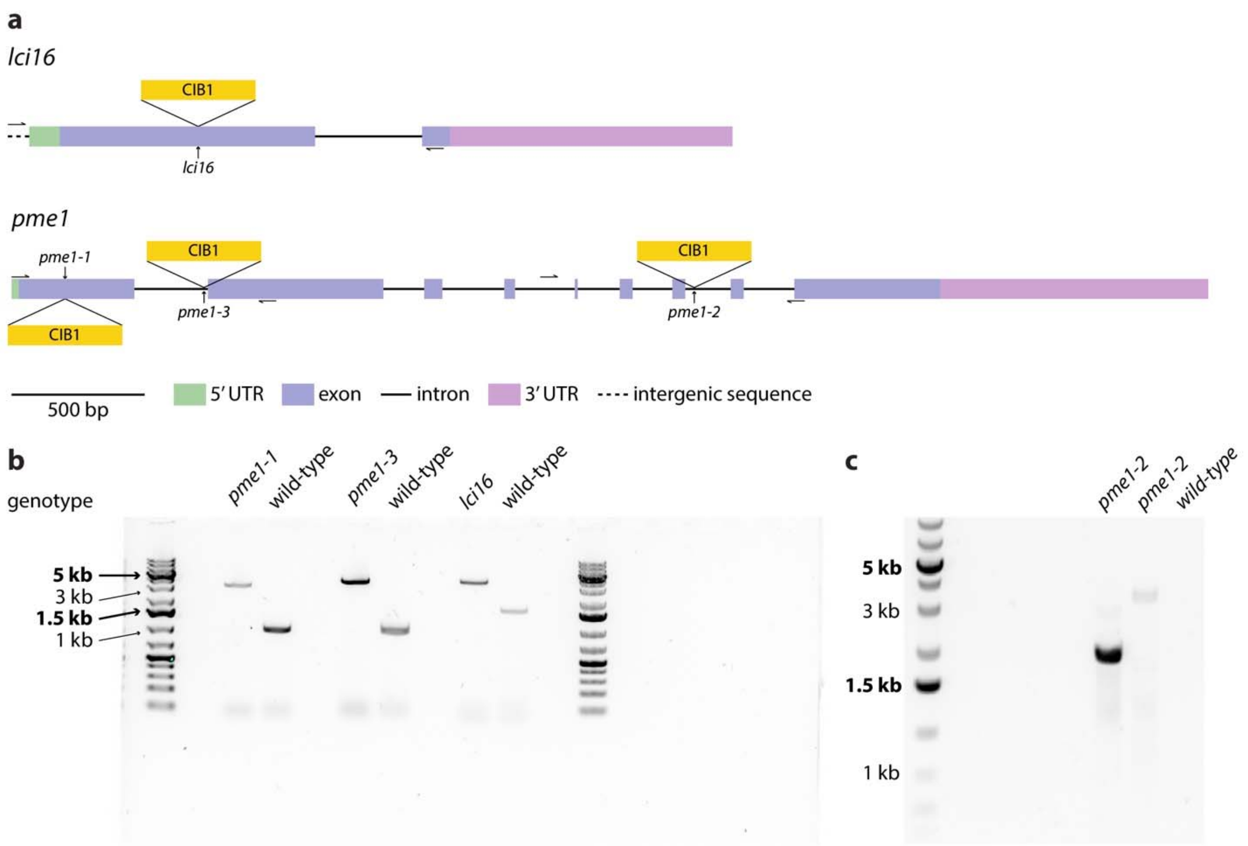

Supporting Information Figure S2: PCR verification of *lci16* and *pme1* insertional mutants

PCR verification of the insertions the *lci16* and *pme1* mutants. (**a**) Maps of the *LCI16* and *PME1* genomic loci showing the locations of the CIB1 insertion cassette and the primers used for PCR verification. The CIB1 cassette and primer sequences are not drawn to scale. (**b-c**) Agarose gels showing amplification across the insertion junction in genomic DNA purified from *pme1-1*, *pme1-3*, and *lci16* (**b**); and *pme1-2* (**c**). Expected amplicon lengths for wild-type gDNA were 1025 bp for *pme1-1* and *pme1-3* using the primers TIP.Z4.For/Rev; 1643 bp for *lci16* using the primers TS.Q.For/Rev; and 1014 bp for *pme1-2* using the primers TIP.Z2/3.For/Rev. For each strain in panel (**b**), the left lane shows the amplicon from the mutant's gDNA and the right lane shows the amplicon from wild-type gDNA using the same primers. Amplicon lengths for each mutant were in excess of 3,000 bp and were longer than the amplicons produced by the same primers in wild-type gDNA, confirming the presence of the insertion in each mutant. In panel (**c**), leftmost lane shows pme1-2 gDNA and a control set of primers, TS.Hb.For/Rev, which amplify a neutral locus at which there is no insertion; and the middle and right lanes show the *pme1-2* insertion junction amplified in *pme1-2* and wild-type gDNA, respectively. There was insufficient wild-type gDNA template to amplify the expected band in the rightmost lane, but the amplicon

bioRxiv preprint doi: https://doi.org/10.1101/2024.10.28.620638; this version posted October 31, 2024. The copyright holder for this preprint (which was not certified by peer review) is the author/funder, who has granted bioRxiv a license to display the preprint in perpetuity. It is made available under aCC-BY 4.0 International license.

from the *pme1-2* gDNA was well in excess of the expected length, confirming the presence of the insertion. Primer sequences can be found in the Materials and Methods section.

bioRxiv preprint doi: https://doi.org/10.1101/2024.10.28.620638; this version posted October 31, 2024. The copyright holder for this preprint (which was not certified by peer review) is the author/funder, who has granted bioRxiv a license to display the preprint in perpetuity. It is made available under aCC-BY 4.0 International license.

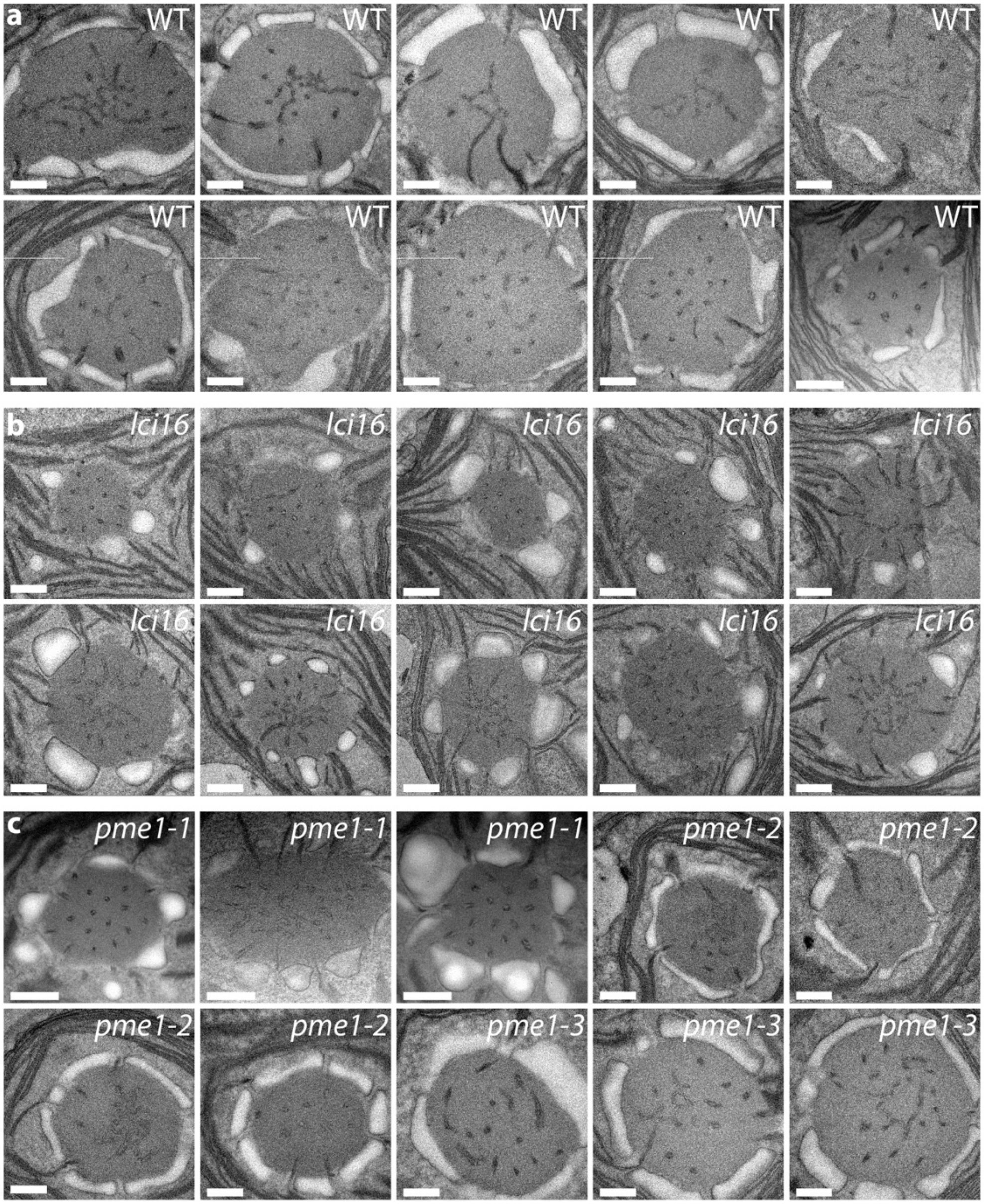

Supporting Information Figure S3: Additional TEM pyrenoid images of insertional mutants of *lci16* and *pme1*

Additional micrographs of wild-type (**a**), *lci16* (**b**), and *pme1* (**c**) pyrenoids, showing that lci16 and pme1 mutant pyrenoids contain central reticulated regions and minitubule-containing peripheral tubules indistinguishable from wild-type cells. Scale bars 500 nm.

bioRxiv preprint doi: https://doi.org/10.1101/2024.10.28.620638; this version posted October 31, 2024. The copyright holder for this preprint (which was not certified by peer review) is the author/funder, who has granted bioRxiv a license to display the preprint in perpetuity. It is made available under aCC-BY 4.0 International license.

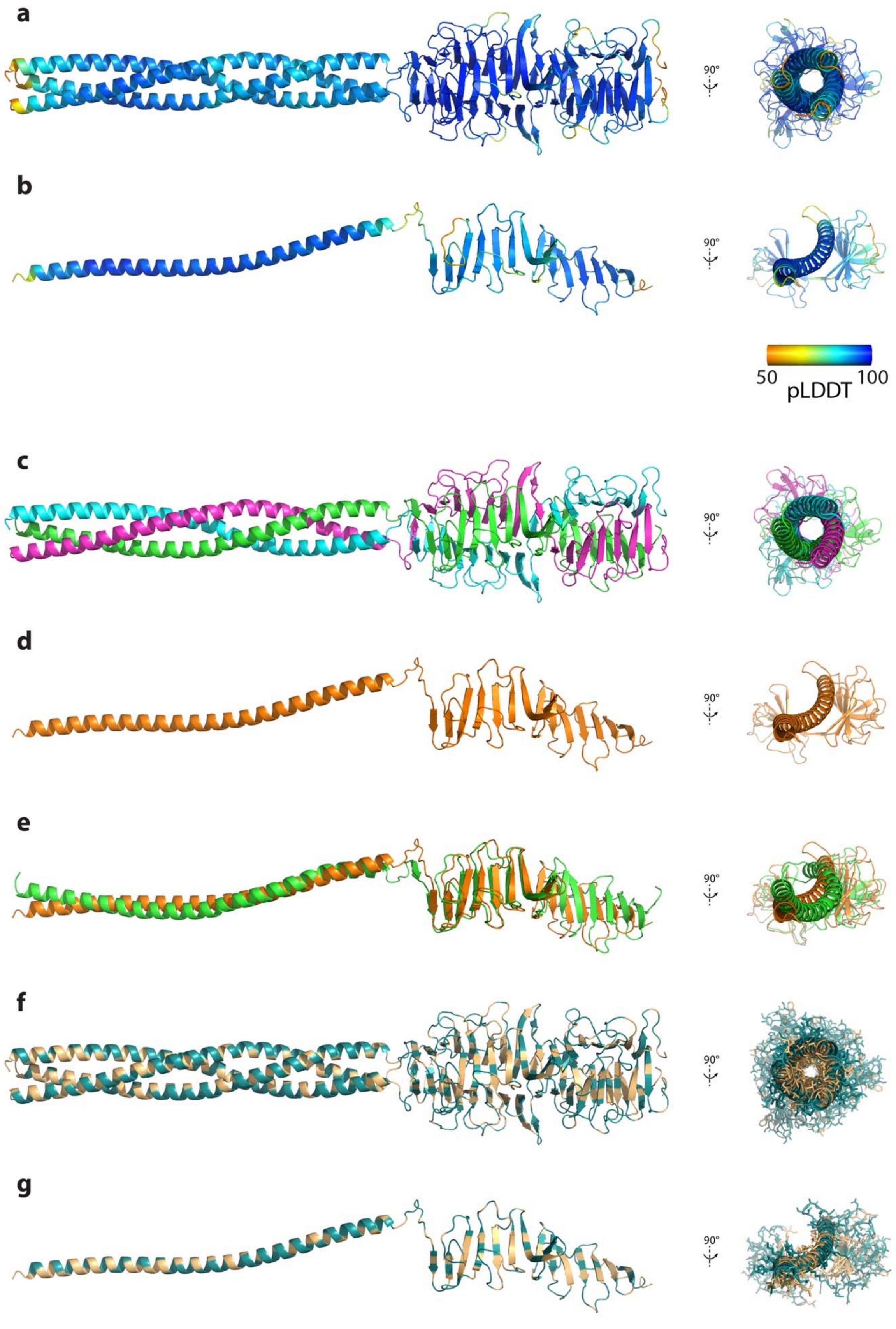

729

bioRxiv preprint doi: https://doi.org/10.1101/2024.10.28.620638; this version posted October 31, 2024. The copyright holder for this preprint (which was not certified by peer review) is the author/funder, who has granted bioRxiv a license to display the preprint in perpetuity. It is made available under aCC-BY 4.0 International license.

Supporting Information Figure S4: Alphafold3 predicts an amphipathic helix and beta sheet in PME1.

Alphafold3 predictions of a trimer **(a,c,f)** and monomer **(b,d,g)** of the residues 380–639 of PME1, the 260 residues at the C-terminus of PME1. **a–b**. The trimer **(a)** and monomer **(b)** colored according to the per-atom confidence metric pLDDT. Confidence scores for the entire structure are higher for the trimer (pTM = 0.83, iPTM = 0.82) than the monomer (pTM = 0.49). **c–e.** Cartoon representation of the extended helix and beta sheet of the predicted trimer **(c)**, in which the helices form a coiled coil and the beta sheets form a beta-barrel-like structure, and the monomer **(d)**, which takes on a similar conformation even in the absence of oligomerization, as shown by an alignment of the monomer with one subunit of the trimer **(e)**. **f–g.** The predicted trimer **(f)** and monomer **(g)** colored by Kyte-Doolittle hydrophobicity (Kyte & Doolittle, 1982), with hydrophilic residues colored deep teal and hydrophobic residues colored beige. In the 90-degree rotations showing the structure along the axis of the helix, side chain visualization has been added to show that the interior of the coiled coil contains almost exclusively hydrophobic residues while the exterior is almost entirely hydrophilic.
